# Shallow Hydrothermal Vent Bacteria and Their Secondary Metabolites with a Particular Focus on *Bacillus*

**DOI:** 10.3390/md19120681

**Published:** 2021-11-29

**Authors:** Revathi Gurunathan, Arthur James Rathinam, Jiang-Shiou Hwang, Hans-Uwe Dahms

**Affiliations:** 1 Department of Medicinal and Applied Chemistry, Kaohsiung Medical University, Kaohsiung 80708, Taiwan; gururevati@gmail.com; 2 Department of Biomedical Science and Environmental Biology, Kaohsiung Medical University, Kaohsiung 80708, Taiwan; 3 Department of Marine Science, Bharathidasan University, Tiruchirapalli 620024, India; 4 Institute of Marine Biology, National Taiwan Ocean University, Keelung 20224, Taiwan; 5 Center of Excellence for Ocean Engineering, National Taiwan Ocean University, Keelung 20224, Taiwan; 6 Center of Excellence for the Oceans, National Taiwan Ocean University, Keelung 20224, Taiwan; 7 Research Center for Environmental Medicine, Kaohsiung Medical University, Kaohsiung 80708, Taiwan; 8 Department of Marine Biotechnology and Resources, National Sun Yat-Sen University, Kaohsiung 80424, Taiwan

**Keywords:** hydrothermal vent (HV), bacteria, extremophile, bioactive substance, molecular phylogeny

## Abstract

Extreme environments are hostile for most organisms, but such habitats represent suitable settings to be inhabited by specialized microorganisms. A marine shallow-water hydrothermal vent field is located offshore in northeast Taiwan, near the shallow shore of the southeast of Kueishantao Island (121°55′ E, 24°50′ N). Research on extremophilic microorganisms makes use of the biotechnological potential associated with such microorganisms and their cellular products. With the notion that extremophiles are capable of surviving in extreme environments, it is assumed that their metabolites are adapted to function optimally under such conditions. As extremophiles, they need specific culture conditions, and only a fraction of species from the original samples are recovered in culture. We used different non-selective and selective media to isolate bacterial species associated with the hydrothermal vent crab *Xenograpsus testudinatus* and the sediments of its habitat. The highest number of colonies was obtained from Zobell marine agar plates with an overall number of 29 genetically distinct isolates. 16S RNA gene sequencing using the Sanger sequencing method revealed that most of the bacterial species belonged to the phylum Firmicutes and the class Bacilli. The present study indicates that hydrothermal vent bacteria and their secondary metabolites may play an important role for the reconstruction of the evolutionary history of the phylum Procaryota.

## 1. Introduction

**Figure 1:**
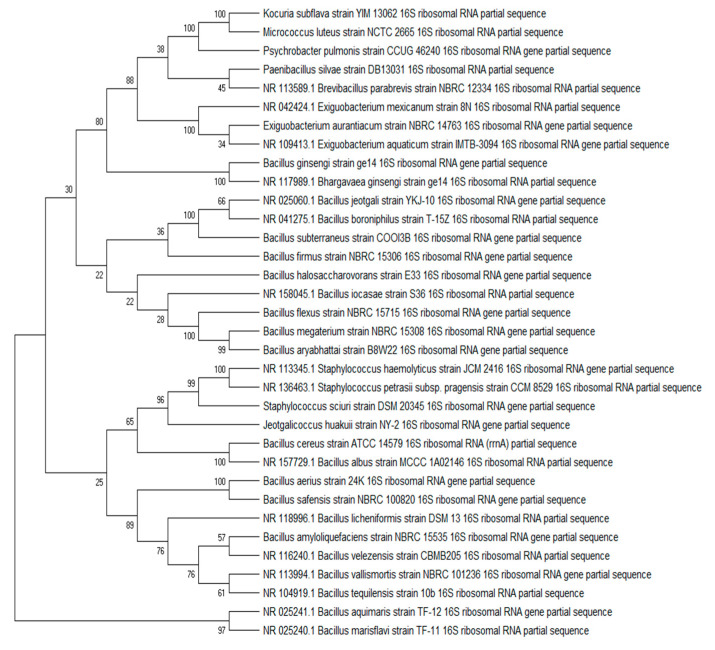
Phylogenetic tree constructed from hydrothermal vent bacteria isolates using MEGA X software with 100 bootstrap values.

Marine and terrestrial environments that are extremely hot are habitats for only a few living organisms. However, such extreme environments present suitable habitats for specialized microorganisms called extremophiles [1]. Many bacteria and archaea are reported from such extremely harsh environments, having the potential to tolerate a wide range of temperatures and pH or high concentrations of salt [2]. Such microbes are called extremophiles and can be further classified on the basis of their specific needs, for example, as thermophiles (adapted to higher temperatures), psychrophilic (adapted to cold temperatures) and halophiles (adapted to high salinities) [3,4]. The interest in studying microorganisms from extreme sites is exemplified by Thomas Brock’s extraordinary discovery of the hot springs of Yellowstone National Park in the 1960s [5,6]. Subsequently, several other thermophilic sites were sampled for thermophilic microorganisms on this planet [7,8]. Kueishan Island, later called Kueishantao (KST), is an island in the southern East China Sea, which erupted about 7000 years ago and is located about 10 km from the Yilan County coast, Taiwan (121°55′ E, 24°50′ N) [2,9,10]. It is located at an intersection between the Okinawa Trough and the Philippine Plate. The eastern sea near KST is a shallow, submarine hydrothermally active area [11,12,13] (Figure 1). About 50 individually discernable hydrothermal vents at depths of 5–30 m were discovered in eastern KST subtidal regions over an area of ~0.5 km^2^ early in the last century [14]. The constant magmatic activity underneath the area of KST is due to the westward expansion of the Okinawa Trough. The KST vent fluids undergo drastic temperature changes that either fall in the range of 78–116 °C or vary down to 30–65 °C [14,15,16,17].

Such shallow-water hydrothermal vents provide few nutrients and, therefore, have an oligotrophic and toxic environment as they are situated near actively bustling coastal or submarine volcanoes [18]. The only multicellular organism found in this region, also considered one of the vent-endemic species living at depths down to 250 m, is the crab *Xenograpsus testudinatus*. This crab predominates in these vents and is able to withstand the sulfur-rich/highly acidic hydrothermal vents with pH values ranging from 1.75 to 4.6. Some recent studies have reported the food requirements of *X. testudinatus*, but only a few reports have focused on the microbes associated with *X. testudinatus* [18,19].

Extremozymes from extremophilic bacteria may have substantial industrial applications as biocatalysts, being functional under harsh treatments, where most of the common enzymes lose their activity and are denatured due to high temperatures or extremes in pH [20]. A wide spectrum of unique properties make them useful in various industrial and other applications [21]. Advanced techniques and methods by which to culture such microorganisms and increase the yield of the production of biomass, enzymes and other biomolecules of interest have been developed [22]. The studies reported so far also support this, and the microorganisms that can withstand harsh and extreme conditions are, therefore, of great interest [23,24].

Hydrothermal vent marine bacteria are difficult to isolate and cultivate because of their particular culture requirements. However, different culture techniques can be optimized in laboratory conditions that make their successful cultivation possible. Such knowledge will also enhance our understanding of the evolution of hydrothermal vent microbiota and their secondary metabolites in this unique extreme environment [25].

The aim of this article was to identify bacterial systematic and metabolic diversity from shallow-water hydrothermal vents. We focus here on media optimization for the isolation of a maximum number of bacterial colonies in order to study their diversity and phylogenetic relationship and the diversity of bioactive compounds. This study focuses on extremophiles since these microbes are expected to tolerate a wide range of, e.g., temperatures, pressure, pH and elevated concentrations of heavy metals. This provides the expectation of harvesting unique secondary metabolites that may also protect the bacteria from extreme physicochemical factors and provide new pathways for energy acquisition.

## 2. Results and Discussion

### 2.1. Bacterial Diversity Studies

Using standard cultivation methods, a maximum number of colonies were isolated using Zobell marine agar and nutrient agar with NaCl as selective media (Table 1). Only a few bacterial colonies can grow on TSA and sea water. Distinct colonies were identified by different morphology and color. Solid bacteriological culture media are mostly used for the isolation and enumeration of bacteria from environmental samples, applying the diluting method where the original bacterial suspension is diluted to the desired folds and a small inoculum is spread over the surface of a solidified medium. The ability of a bacterium to grow and yet fail to show up as a CFU through the plate or broth culture method leads to “viable but non-culturable” (VBNC) bacteria [26]. The “unculturable state” discussed here is tentatively defined as the physiological condition in which marine bacteria do not grow, e.g., as colonies (CFUs) on agar plates [27]. The VNC concept has a broad impact for the majority of research aspects in environmental microbiology [28]. Some of the bacterial cells retrieved from environmental samples may simply die during the cultivation process. Pasotti et al. [29] reported that some of the lethal effects are associated with physical or chemical agents, which lead to a process of “self-destruction” by the cells themselves.

**Figure 2:**
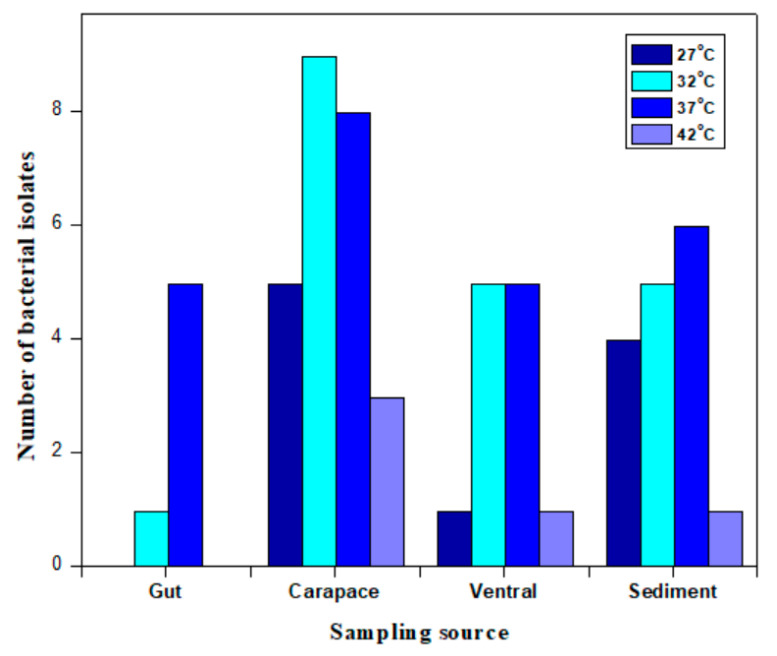
Bacterial diversity according to 16S RNA from different sediment and biogenic crab substrates.

The sequencing results revealed that most of the bacterial species isolated by Gurunathan et al. (2021) belonged to the phylum Firmicutes. From the phylum Proteobacteria, one isolate was identified as *Psychrobacter pulmonis* belonging to the phylum Proteobacteria, order Pseudomonadales. From the phylum Actinobacteria, two bacterial colonies were isolated. One bacterium was identified as *Kocuria subflava*, belonging to the family Micrococcaceae. The other isolate was identified as *Micrococcus* belonging to the family Micrococcaceae and the species *M. luteus*. The predominant bacteria belonged to the phylum Firmicutes, family Bacillaceae. The different isolated species of *Bacillus* were *B. aerius*, *B. aryabhattai*, *B. ginseng*, *B. aquimaris*, *B. iocasae*, *B. firmus*, *B. marisflavis*, *B. safensis*, *B. subterraneus*, *Micrococcus luteus*, *Bacillus megaterium*, *Bacillus cereus*, *B. vallismortis*, *B. licheniformis*, *B. jeotgali*, *Bacillus boroniphilus*, *B. halasaccharovorans*, *Bacillus flexus*, *Bacillus velezensis*, *B. albus*, *B. tequilensis*, *B. paramycoides* and *Brevibacillus parabrevis.* Five bacterial isolates belonged to different genera and species of the taxon Bacillaceae, i.e., *Paenibacillus silvae*, *Exiguobacterium aquaticum*, *Exiguobacterium aurantiacum*, *Staphylococcus sciuri* and *Jeotgalicoccus huakuii.* The phylogenetic tree was built using BLAST and MEGA software version X (Figure 1). The sampling source provided a difference since the bacterial colonies isolated from different samples were different. A maximum of colonies were isolated from the HV crab’s carapace and its ambient sediments (Figure 2).

Related to our studies was the bacterial diversity of hot springs from Araró, Mexico, identified by 16S rDNA sequencing [30]. Seventeen bacteria were isolated and identified from both hot springs, which included bacteria from the phylum Firmicutes, Proteobacteria and Actinobacteria, and Firmicutes strains from *Paenibacillus*, Bacillus and Exiguobacterium (76%), Pseudomonas and Aeromonas (18%) from (Proteobacteria) and 6% of Actinobacteria with the genus Microbacterium. The 16S rDNA sequences indicated *Bacillus* as the dominant group including *Bacillus subtilis*, *B. licheniformis*, *B. pumilus* and *B. cereus*. The most frequently occurring species were *Bacillus amyloliquefaciens*, *Aeromonas hydrophila*, *Bacillus vietnamensis*, *Bacillus megaterium*, *Bacillus boroniphilus* and *Exiguobacterium profundum*. The bacterial species appearing in only one of the samples were *Exiguobacterium sibiricum*, *Bacillus oceanisediminis*, *Paenibacillus favisporus*, *Paenibacillus pabuli*, *Pseudomonas psychrotolerans*, *Pseudomonas stutzeri* and *Microbacterium oleivorans* [30]. The results reported by Prieto et al. [30] had similar findings to those of our study. The species percentage as reported by the above authors was similar to that of our studies where *Bacillus* was dominant and only one species of *Paenibacillus* was found. The microbial diversity studied by Cui et al. [31] in the South China Sea from two cold seep systems in gas-hydrate-bearing sediments showed a relatively low abundance of Planctomycetes and Firmicutes bacteria. When the authors applied the Mann–Whitney U test, they found among the phylum Firmicutes Bacilli and Clostridia, Bacteroidia from the phylum Bacteroidetes, Acidobacteria and Cyanobacteria in abundance in the non-gas hydrate zone [31]. The microbial isolates studied by Kumar et al. [32] showed diversity in subsurface seawaters from the western coastal ecosystem of the Arabian Sea, India. The bacterial communities were dominated here by Proteobacteria, followed by Firmicutes [32]. Representatives of *Bacillus*, known for their tolerance, were predominant in most extreme environments. The bacteria in our study were isolated from sulfur-rich hydrothermal vents. Meyer-Dombard et al. [33] studied the microbial diversity at an arsenic- and iron-rich vent with biogeochemical cycling carried out using bacteria at Tutum Bay from Papua in New Guinea. The bacterial isolates were classified as alpha- and beta-Proteobacteria, and the genera were *Thermus* and *Pyrobaculum* [33]. In the bacterial phylogenetic study of Shinde et al. [34], 49 newly generated sequences within four bacterial phyla from tarballs were collected from Betul, Goa, India, which included Proteobacteria (38 species), Gammaproteobacteria (31 species), Alphaproteobacteria (7 species), Actinobacteria (6 species), Firmicutes (4 species) and Bacteroidetes (1 species). Among the Actinobacteria, the species belonged to *Cellulomonas* (three species) and *Brevibacterium* (two species); from the Firmicutes, *Bhargavaea* (three species); and only one species was found from *Bacillus* (one species). In a study from southeastern Arabian Sea sediments, the dominant genera were *Clostridium* and *Bacillus* belonging to the phylum Firmicutes [35]. The other genera were *Lactobacillus*, *Enterococcus*, *Oscillospira* and *Staphylococcus* [35]. Isolates from the Wadden Sea (North Sea) revealed that 16% of the isolates were affiliated to the *Bacillus/Clostridium* group, and 10 % belonged to the phylum Actinobacteria. The presence of a relatively high number of Gram-positive bacteria may indicate the specific characteristics of the Wadden Sea environment [36].

Both abundance and bacterial diversity are generally substantially underestimated by CFU counts. The present study substantiated this notion by identifying 31 different bacterial strains through a comparison of 16S rRNA gene sequences from various microbial isolates from hydrothermal vent sediments and the invertebrate crab *Xenograpsus testudinatus.*

### 2.2. Characteristics of the Isolated Bacterial Colonies

Culture characteristics were studied for isolated bacterial colonies. The morphologies and phenotypes were observed. The results showed that most of the bacteria belonging to the genus *Bacillus* have white colonies, such as *Bacillus albus*, and cream-colored colonies, such as *Bacillus flexus*, although a few species showed pigmented colonies. The color of the colonies varied; in *Bacillus tequilensis*, the colonies appeared as yellow; the species *Micrococcus luteus* appeared as bright yellow; and the colonies of *Bacillus jeotgali* and *Brevibacillus parabrevis* appeared as pale yellow. Some colonies had an orange color, such as *Exiguobacterium mexicanum* and *Exiguobacterium aurantiacum*. Apart from the color differences, some colonies appeared glossy, such as *Paenibacillus silvae* and *Bacillus firmus*; colonies of *Bacillus amyloliquefaciens* showed highly raised, mucous filled colonies. *Bacillus licheniformis* showed branched, hair-like, irregular morphology, and *Bacillus cereus* showed branched, fuzzy, irregular edges (Table 2).

The number of bacterial colonies obtained from the carapace was higher than compared to other sources. The sampling source provided a difference since the bacterial colonies isolated from different samples were different. A maximum of colonies were isolated from HV crab carapace and sediment from its habitat (Figure 2). Additionally, the bacterial species characterized from the crab carapace (*n* = 11) and sediments were able to adapt to temperatures ranging at least between 27 and 42 °C. From the ventral side of crabs, the number of obtained colonies was fewer, and the optimum temperature at which bacterial species could grow was between 32 °C and 37 °C.

### 2.3. Products from Marine Isolates

**Figure 3:**
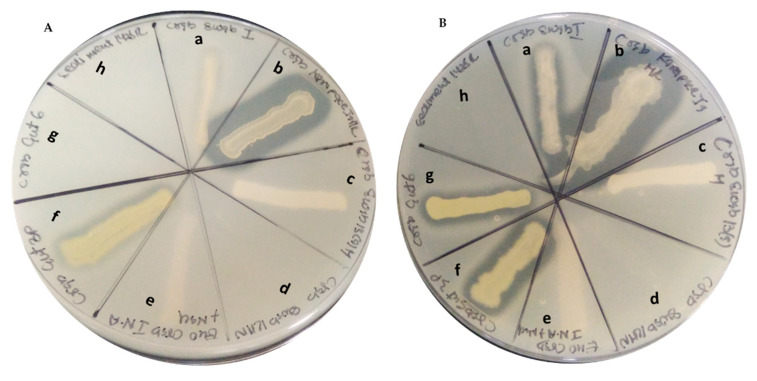
Protease assay. (**A**). In the presence of 500 µM ZnSO_4_. (**B**). Protease assay lacking ZnSO_4._ (a. *Bacillus licheniformis*, b. *Bacillus amyloliquefaciens*, c. *Staphylococcus haemolyticus*, d. *Bacillus jeotgali*, e. *Bacillus firmus*, f. *Bacillus aquimaris*, g. *Micrococcus luteus*, h. *Bacillus subterraneus*).

Thermophilic bacteria associated with hydrothermal vents are promising sources for research and commercial applications. In our study, 29 bacterial strains were isolated and cultivated from shallow-water marine hydrothermal vents around Kueishantao for the screening of protease activity. The protease assay was performed with and without ZnSO_4_ (Figure 3). Different concentrations of this metal ion were studied for its effect on protease activity (Table 3). The results demonstrate that *Bacillus licheniformis*, *Bacillus amyloliquefaciens*, *Bacillus aquimaris* and *Micrococcus luteus* hydrolyze the protein. The zone of lysis was observed for these isolates. In the presence of ZnSO_4,_ the activity was concentration dependent. With low concentrations of ZnSO_4,_ the protease activity was high, whereas protease activity was inhibited in the presence of high concentrations of ZnSO_4_ (Figure S1).

The strain producing the highest activity was identified as *Bacillus cereus*. The gene for protease was amplified from *Bacillus cereus*, cloned and expressed in *Escherichia* *coli* *BL21* cells. The protein sequence retrieved from mass spectrometry analysis showed sequence similarity to that of the subtilisin-like serine protease protein, belonging to the family of S8 peptidases with a molecular weight of about 38 kDa and a 38 aa signal peptide region. SLSP-k is a monomeric protein that is active over wide temperature (40–80 °C) and pH (7–11) ranges, showing maximal hydrolytic activity at pH 11 and at a temperature of 50 °C. The proteolytic activity was further elevated by Co^2+^, Ca^2+^, and Mn^2+^, DTT, and inhibited by Cu^2+^, Cd^2+^, Fe^2+^, PMSF, and EDTA. SLSP-k was observed to be stable in non-anionic and anionic solvents and detergents. This protein shows keratinolytic degradation of chicken feathers. Hence, the protein is suitable for research applications, industrial product making and waste management [4].

A novel thermostable exopolysaccharide (EPS-B3-15) isolated from the thermophilic marine *Bacillus licheniformis* (B3-15) composed of glucose and mannose was recently reported as a beneficial compound with applications in bio- and nanotechnology, material sciences and pharmacology since it showed thermostability at high temperatures [37]. Although several bacterial xylanases were studied, only few could successfully be extracted from marine-associated micro-organisms. Khandeparker et al. (2011) [38] isolated *Bacillus subtilis* cho40 and characterized a novel halotolerant xylanase from Chorao Island near Goa, India. This could be used for bioethanol production from marine seaweeds. The extracellular xylanase was produced by solid-state fermentation (SSF) with wheat bran as a carbon source. The optimal temperature was 60 °C, and the optimal pH was reported as 6.0. Xyn40 was also reported as halophilic and showed maximum activity at 0.5 M NaCl. The cloned xylanase gene, *xyn40*, was 645 bp long and coded for 215 amino acids with a molecular size of 22.9 kDa.

Shofiyah et al. [39] extracted α-amylase from *Bacillus megaterium* NL3, which was symbiotic with a cnidarian from Lake Kakaban, India. Shofiyah et al. [39] cloned the α-amylase-encoding gene BmaN2 and expressed it in *E. coli* (DE3). This α-amylase exhibited the highest activity at a temperature of 60 °C and a pH of 6. Its specific activity was calculated as 28.7 U mg^−1^. The enzyme activity was highly upregulated by Ca^2+^ and decreased in the presence of EDTA. BmaN2 also showed high catalytic activity on soluble starch with *k*_cat_*/K*_M_ values of 14.1 mL mg^−1^s^−1^. Although the most preferred substrate with maximum hydrolytic activity by BmaN2 was granular wheat, BmaN2 can also hydrolyze raw starch, such as that of potato, sago, wheat, canna, corn, rice and cassava. An L-Asparaginase-producing bacterium, *Bacillus velezensis*, was isolated from marine sediments by Mostafa et al. [40]. l-Asparaginase free from glutaminase activity is required for medical applications; however, it also shows severe side effects. The purified enzyme was 39.7 KDa, as predicted by SDS, and it was efficiently active at pH 7.5 and a temperature of 37 °C. The enzyme was studied for its anticancer properties in breast adenocarcinoma cell lines, and the results showed significant activity towards MDA-MB-231 cells with IC_50_ values of 12.6 ± 1.2 μg/mL as compared to MCF-7 cells with IC_50_ values of 17.3 ± 2.8 μg/mL.

Hamiche et al. [41] isolated *Bacillus amyloliquefaciens* (S13) from the phaeophycean macroalga *Zonaria tournefortii*. This bacterium produced extracellular moderately elastolytic and keratinolytic enzymes. The study suggested that the present enzymes were monomers, designated as KERZT-A of 28 kDa and KERZT-B 47 of kDa. The NH_2_-terminal amino acid showed high sequence homology with keratinases from *Bacillus*. Whereas KERZT-A showed maximal activity at 50 °C and pH 6.5, KERZT-B showed highest activity at 60 °C and pH 8. Both enzymes were completely inhibited by PMSF (phenylmethanesulfonyl fluoride) and DFP (diiodopropyl fluorophosphate), indicating that they belong to the serine keratinase family. Additionally, the keratinase KERZT-A displayed a higher level of specificity towards substrate hydrolysis. Moreover, the catalytic efficiency was higher than that of KERUS from the *Brevibacillus* *brevis* strain US575, NUE 12 MG (commercial enzyme) and KERZT-B, an unhairing keratinase. Thus, the enzyme is of interest for medicinal applications as well as for the cosmetic and the leather industries.

In the search of substitutes for synthetic plastics, Mohandas et al. [42] screened, isolated and identified polyhydroxylalkanoates (PHAs) produced by bacteria isolated from marine water samples. A potent isolate of the moderately halophilic *Bacillus cereus* (MCCB 281) produced PHA co-polymers with glycerol as the main carbon source under optimized processing parameters involving a central composite design for increased PHA fermenter production. It showed a 1.5-fold higher PHA yield. PHA nanoparticles with an approximate size of 179 nm were created for medical purposes, and biocompatibility testing was carried out with L929 mouse fibroblast cell lines. Al Farraj et al. [43] enhanced the fibrinolytic enzyme (32 kDa) yield from a marine *Bacillus flexus* by 3.5-fold using a central composite design standardized culture medium with a high enzyme yield (4711 ± 29.3 U/g of substrate). The fibrinolytic protein was expressed to its maximum at 50 °C and a pH of 8.

For the decontamination of organic pollutants, 19 bacterial isolates secreting extracellular enzymes were examined for their solvent tolerance by Trivedi et al. [44]. *Bacillus aquimaris* produced a cellulase with optimal activity at pH 11 and at 45 °C. The enzyme was functionally stable and showed activity even at higher temperatures (75 °C) and at pH 12. Enzyme activity in the presence of different metals was highest with Co^+2^ followed by Fe^+2^ and NaOCl_2_. In the presence of CuSO_4_, the activity was high as compared to KCl and NaCl. The enzyme stability reported with the addition of solvents was highest in benzene (122%), followed by methanol (85%), acetone (75%) and toluene (73%), and lowest with heptane (42%). The activity of the enzyme was further enhanced when the protein was pre-incubated in ionic solvents such as 1-ethyl-3-methylimidazolium methane sulfonate and 1-ethyl-3-methylimidazolium bromide by 155% and 150%, respectively.

One of the HPLC fractions of a biosurfactant was extracted from the marine *Bacillus circulans* by Das et al. [45], showing maximum surface tension-reducing properties and pronounced antibacterial action (MIC, MBC) against a group of Gram-positive and Gram-positive and -negative bacterial pathogens and a few multidrug-resistant (MDR) pathogenic but non-hemolytic clinical bacteria. It was also reported that only one of the HPLC fractions showed antimicrobial efficacy and was non-hemolytic from crude biosurfactants [45].

He et al. [46] used preparative high-speed counter-current chromatography (HSCCC) to study the two macrolactin antibiotics from the marine bacterium *Bacillus amyloliquefaciens.* Previously, several reports showed that bioactive secondary metabolites can be obtained from sponge-associated bacteria. Kiran et al. [47] isolated 56 bacteria from the marine sponge *Callyspongia diffusa* and analyzed them for their activity against the MDR *Staphylococcus aureus* strain. The 16S rRNA sequence and phylogenetic analysis identified the bacteria as *Bacillus tequilensis* showing antimicrobial activity. The isolate MSI45 was proven to produce the novel antibacterial compound pyrrolo [1,2-a] pyrazine-1,4-dione, hexahydro. This compound showed significant inhibition against multidrug-resistant *Staphylococcus aureus* with an MBC of 20 ± 0.072 mg L^−1^ and an MIC of 15 ± 0.172 mg L^−1^. The compound showed high antioxidant activity and was non-hemolytic. Saggese et al. [48] stated that the marine strain *Bacillus pumilus* (SF214) produced a protein compound with antibacterial activity. The small molecule of 3 kDa exhibited activity against *Staphylococcus aureus* and the other larger molecule of 10 kDa was active against the pathogen *Listeria monocytogenes.* A mass spectrometry analysis classified the small molecule as pumilacidin, a non-ribosomally synthesized biosurfactant that is active against *Staphylococcus*. Nisha et al. [49] characterized exopolysaccharides (EPS) produced by the marine bacterium *Micrococcus luteus* isolated from a ship hull at Cochin port, India. An EPS-associated compound from *M. luteus* was isolated and characterized, and the results showed antibacterial and antifungal activities against pathogens. An in vitro assay for cytotoxic activity of the pigment indicated antibacterial and antifungal activity. Furthermore, UVA rays’ potential to absorb the pigment makes it unstable when applied in sunscreens.

### 2.4. The Identification of Differences in Bacterial Colony Occurrence from the Sampling Source

**Figure 4:**
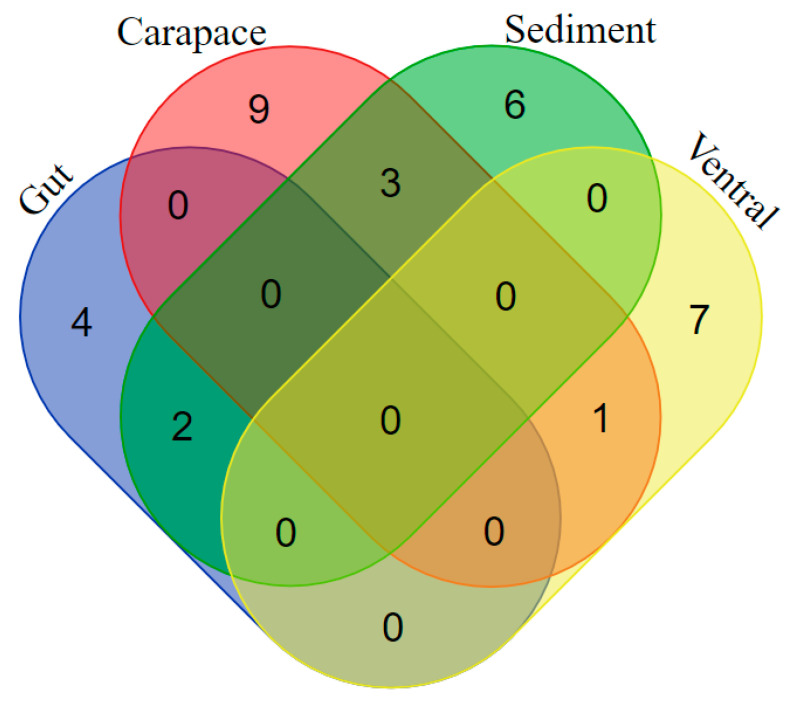
Venn diagram depicting the species commonly found at the sampling source, including unique species. Subset 1: *Bacillus aerius*; Subset 2: *Bacillus ginseng* and *Bacillus aryabhattai*; Subset 3: *Exiguobacterium aurantiacum*, *Bacillus cereus* and *Bacillus albus*; Subset 4: *Kocuria subflava*, *Paenibacillus silvae*, *Bacillus tequilensis* and *Micrococcus luteus*; Subset 6: *Bacillus subterraneus*, *Brevibacillus parabrevis*, *Bacillus marisflavis*, *Bacillus velezensis*, *Jeotgalicoccus huakuii* and *Bacillus megaterium;* Subset 7: *Staphylococcus sciuri*, *Bacillus flexus*, *Bacillus halosaccharovorans*, *Bacillus jeotgali*, *Bacillus licheniformis*, *Bacillus amyloliquefaciens* and *Staphylococcus haemolyticus;* Subset 9: *Bacillus firmus*, *Bacillus safensis*, *Bacillus iocasae*, *Bacillus aquimaris*, *Bacillus marisflavis*, *Bacillus vallismortis*, *Exiguobacterium mexicanum* and *Psychrobacter pulmonis*.

The bacteria restricted to the gut of the crab *Xenograpsus testudinatus* were *Kocuria subflava*, *Paenibacillus silvae*, *Bacillus tequilensis* and *Micrococcus luteus*; those restricted to sediments were *Bacillus subterraneus*, *Brevibacillus parabrevis*, *Bacillus marisflavis*, *Bacillus velezensis*, *Jeotgalicoccus huakuii* and *Bacillus megaterium*; those restricted to the ventral crab plate were *Staphylococcus sciuri*, *Bacillus flexus*, *Bacillus halosaccharovorans*, *Bacillus jeotgali*, *Bacillus licheniformis*, *Bacillus amyloliquefaciens* and *Staphylococcus haemolyticus;* and those from the carapace (=dorsal plate) were *Bacillus firmus*, *Bacillus safensis*, *Bacillus iocasae*, *Bacillus aquimaris*, *Bacillus marisflavis*, *Bacillus vallismortis*, *Exiguobacterium mexicanum* and *Psychrobacter pulmonis*. The species common to the gut and sediment were *Bacillus ginseng* and *Bacillus aryabhattai*. The bacteria found both in the sediments and on the carapace were *Exiguobacterium aurantiacum*, *Bacillus cereus* and *Bacillus albus*. *Bacillus aerius* was isolated from both the ventral plate and the carapace (Figure 4).

The reports of the bacterial diversity of the blue crab *Callinectes sapidus* from the Gulf of Thermaikos, North Aegean Sea, Greece, showed that it comprised Proteobacteria and Tenericutes, followed by Actinobacteria and Firmicutes. Firmicutes were found in abundance in crabs when stored at 4 °C. Additionally, species of *Psychrobacter* spp. were isolated from crab samples [50]. In another study, culturable microbiota from *Chionoecetes opilio*, *Chionoecetes* sp. and *C. japonicus* were isolated using six different media. The phylogenetic relation obtained by 16S rRNA gene sequences showed that the culture belonged to *Acinetobacter*, *Pseudomonas* and *Stenotrophomonas.* In the crab *Chionoecetes opilio*, *Bacillus* spp. were localized in the heart and in the gill, as in *Chionoecetes opilio* and *Chionoecetes japonicus. Bacillus* spp. was isolated from carapace and from *Chionoecetes* sp.; *Bacillus* spp. were identified from carapace juices and the heart [51]. An analysis of the bacterial diversity of the crab *Callinectes sapidus* revealed that the predominant bacterial genus present in the hemolymph was *Vibrio*, followed by *Bacillus*, *Acinetobacter* and *Flavobacterium* [52,53].

## 3. Materials and Methods

### 3.1. Sample Collection and Colony Isolation

Crab *(Xenograpsus testudinatus*) and sediment samples from Kueishantao hydrothermal vents were aseptically collected and immediately processed after bringing them to the laboratory. Different media were prepared to isolate a maximum number of individual bacterial species. The media used were Nutrient agar, Luria Bertani agar, Tryptic soy agar, TCBS media, Zobell marine media, brain heart infusion agar, sea water complete and Casamino acid sea water. All the media were prepared in either sea water or distilled water. From the crab samples, swabs were prepared from the crab dorsal, ventral portion and gut and were spread on all the respective media plates. The crab samples were homogenized and serially diluted up to 10^5^. The sediment samples were serially diluted to 10^5^, and 100 µL from each dilution was spread on all the different media plates. The plates were incubated at 27 °C, 32 °C, 37 °C and 42 °C.

### 3.2. DNA Sequencing and Phylogenetic Analysis

A single colony was inoculated in the respective medium, which showed the optimal growth of the bacteria at the optimal temperature and was kept in a shaking incubator at 150 rpm. Genomic DNA was isolated applying a GeneJET Genomic DNA Purification Kit (Thermo Scientific, Waltham, MA, USA). The PCR reaction with Taq buffer (1X), dNTP (0.2 mM), reverse and forward primers (1 µM each), 50–100 ng template DNA, Taq DNA polymerase (1.25 U) (New England biolabs) and bi-distilled water to a final volume of 25 µL was set. Universal primers, 27F (5′-AGAGTTTGATCCTGGCTCAG-3′) and 1492R (5′-GGTTACCTTGTTACGACTT-3′) were used to amplify the 16S rRNA sequence. The PCR reaction conditions were set with initial denaturation of 94 °C for 5 min, 35 cycles of 95 °C (1 min), 55 °C (1 min), 72 °C (1:40 min) and final elongation at 72 °C (10 min), and the amplified PCR product was run in agarose gel and purified by a GeneJet PCR purification kit (Thermo Scientific, Waltham, MA, USA). The eluted DNA samples were sent for 16S rRNA sequencing.

### 3.3. Phylogenetic Tree

For 16S rRNA sequencing, both forward (27F) and reverse (1492R) primers were used. Sequences were obtained by Sanger sequencing. Forward and reverse sequences were analyzed and assembled using MEGAX [54]. The full-length sequences were used for nucleotide sequence analysis to find highly similar species [55]. Phylogenetic trees were built using MEGAX [54]; sequence alignment using Muscle tool was performed; and tree estimates were retrieved using the maximum likelihood test (100 bootstraps).

### 3.4. Protease Assay

A protease assay was performed using skim milk powder. Skim milk agar plates were prepared using 1% skim milk powder. The bacterial colonies were inoculated in broth and allowed to grow overnight. The overnight culture was streaked on pre-prepared agar plates and incubated at 37 °C again overnight. A zone of lysis was observed. A study was performed with and without the presence of ZnSO_4._ Different concentrations of ZnSO_4_ were used to study its effect on protease activity.

## 4. Conclusions

Using different cultivation methods, a maximum number of colonies were isolated, and distinct colonies were classically identified by different morphology or color. The present study is the first report of microbial diversity derived from laboratory cultures that were classified by sequencing. The sequencing results revealed that most of the bacterial species belonged to the phylum Firmicutes. The classified bacteria mostly belonged to the genus *Bacillus*. The number of bacterial colonies obtained from the carapace was higher than compared to other sources. Bacterial colonies differed according to sample origin. The highest number of colonies was isolated from HV crab carapace and sediments. This study was carried out to derive bacteria from extreme conditions with the ability to tolerate a wide range of pH, high temperatures, varying pressure and nutrient availability. This provides the expectation of extracting unique metabolites that protect organisms from extreme physicochemical factors. Additionally, this can be used to study the pathways adopted by such extremophiles to adapt to toxic compounds and physical conditions in such extreme environments. Microbial adaptations at the genomic, proteomic and metabolomic level are expected to be of substantial applied interest for biotechnology, medical and industrial applications.

## Figures and Tables

**Table 1:** Colony forming units of bacterial colonies obtained from different media.

S. No.	Media	No. of Different CFUs Obtained
1	Nutrient media supplemented with NaCl	7
2	Nutrient media without NaCl	12
3	Tryptic soy media	8
4	Zobell marine media	18
5	Seawater complete	2
6	Casamino acid seawater	2

**Table 2:** Bacterial phenotypes studied here and their optimal temperature and media.

No.	Bacteria	Optimal Media	Temperature (°C)	Phenotype
1	*Bacillus aerius*	Lb without and with NaCl, NA, ZMA	30–37	White, irregular, raised
2	*Staphylococcus haemolyticus*	NA, LB, TSA	37	Light yellow
3	*Bacillus aryabhattai*	MA, Nutrient and Lb with NaCl	37	Opaque, white, raised, irregular
4	*Bacillus ginsengi*	NA, LB, TSA	37	Light yellow
5	*Bacillus aquimaris*	MA, Nutrient and Lb with NaCl	30–37, 42	Yellow pale, circular to slightly irregular and raised
6	*Bacillus iocasae*	Nutrient agar with 2% NaCl, Zobell marine agar	32–37	Off-white
7	*Bacillus firmus*	TSA, LB, NB with NaCl, MA	37	Shiny, circular, semi-transparent, flat colonies
8	*Kocuria subflava*	MA	37	Yellow colonies
9	*Bacillus marisflavis*	Marine agar, LB with and without NaCl	27	Pale yellow, smooth, circular to slightly irregular
10	*Bacillus safensis*	TSA, with and without NaCl	32–37, 42	Irregular margins, off-white/cream
11	*Exiguobacterium aurantiacum*	MA	27–37,42	Orange colonies, flat
12	*Bacillus subterraneus*	TSA	37	Transparent, irregular colonies
13	*Staphylococcus sciuri*	TSA, NA, MA	37	Yellow, circular
14	*Micrococcus luteus*	NA with NaCl, MA	37	Light yellow, regular circular colony
15	*Bacillus megaterium*	NA without and with NaCl	32	Foamy white colonies, irregular
16	*Jeotgalicoccus haukii*	MA, NA with NaCl	37	Round, smooth, circular, white
17	*Bacillus cereus*	MA, LB, NA	27–32	Irregular, opaque, white cream, little fuzzy appearance
18	*Brevibacillus parabrevis*	Trypticase soy agar, MA	32–37	Pale yellow, circular
19	*Bacillus vallismortis*	NA, MA	27–32	Opaque, smooth circular
20	*Psychrobacter pulmonis*	MA	37	White and opaque
21	*Bacillus licheniformis*	MA, LB, NA	27–42	Opaque, hair-like outgrowths, whitish, colonies round to irregular
22	*Paenibacillus silvae*	MA, NA	32	Shiny, smooth, beige color, irregular
23	*Exiguobacterium mexicanum*	MA	37	Orange, circular
24	*Bacillus jeotgali*	MA, NA 2% NaCl	32	Smooth and flat, irregular colony, cream yellow to light yellow
25	*Bacillus boroniphilus*	MA, NA, LB 2% NaCl		Opaqe, smooth surface
26	*Bacillus halasaccharovorans*	MA	32	Circular, smooth, creamy
27	*Bacillus flexus*	10% NaCl, 2%NaCl, MA lb	32	Creamish white, smooth and opaque
28	*Bacillus velezensis*	Tryptic soy agar (TSA or tryptic soy broth (TSB)	27	Creamish white, irregular
29	*Bacillus albus*	LB medium with and without NaCl NA MA	32	White regular
30	*Bacillus tequilensis*	TSA, MA	37	Round, smooth, yellowish in colour
31	*Bacillus amyloliquefaciens*	MA	37	Irregular, raised, white mucous, filled and sticky

**Table 3:** Bacterial protease assay using skim milk in the presence of different concentrations of ZnSO_4_ (+: zone of inhibition is present; −: zone of inhibition is not present).

S. No.	Bacterial Strain	Protease Assay in the Presence of ZnSO_4_				
		50 µM	100 µM	500 µM	1 mM	10 mM
1	*Bacillus amyloliquefaciens*	+	+	+	+	−
2	*Bacillus aquimaris*	+	+	+	+	−
3	*Micrococcus luteus*	+	+	−	−	−
4	*Bacillus jeotgali*	−	−	−	−	−
5	*Bacillus firmus*	−	−	−	−	−

## Supplementary Materials

The following are available online at https://www.mdpi.com/article/10.3390/md19120681/s1, Figure S1. Protease assay in the presence of 50 µM ZnSO4. B. Protease assay with 100 µM ZnSO4. C. Protease assay with 1 mM ZnSO4. D. Protease assay with 10 mM ZnSO4 (a. *Bacillus licheniformis*, b. *Bacillus amyloliquefaciens*, c. *Staphylococcus haemolyticus*, d. *Bacillus jeotgali*, e. *Bacillus firmus*, f. *Bacillus* aquimaris, g. *Micrococcus luteus*, h. *Bacillus subterraneus*).
