# Supplementary material for: Shallow Hydrothermal Vent Bacteria and Their Secondary Metabolites with a Particular Focus on Bacillus
Source: Mar Drugs. 2021 Nov 29;19(12):681. doi: 10.3390/md19120681 (PMC8704404; doi:10.3390/md19120681)
Supplement: Supplementary file 1 [file marinedrugs-19-00681-s001.zip › marinedrugs-1418930-supplementary.pdf]

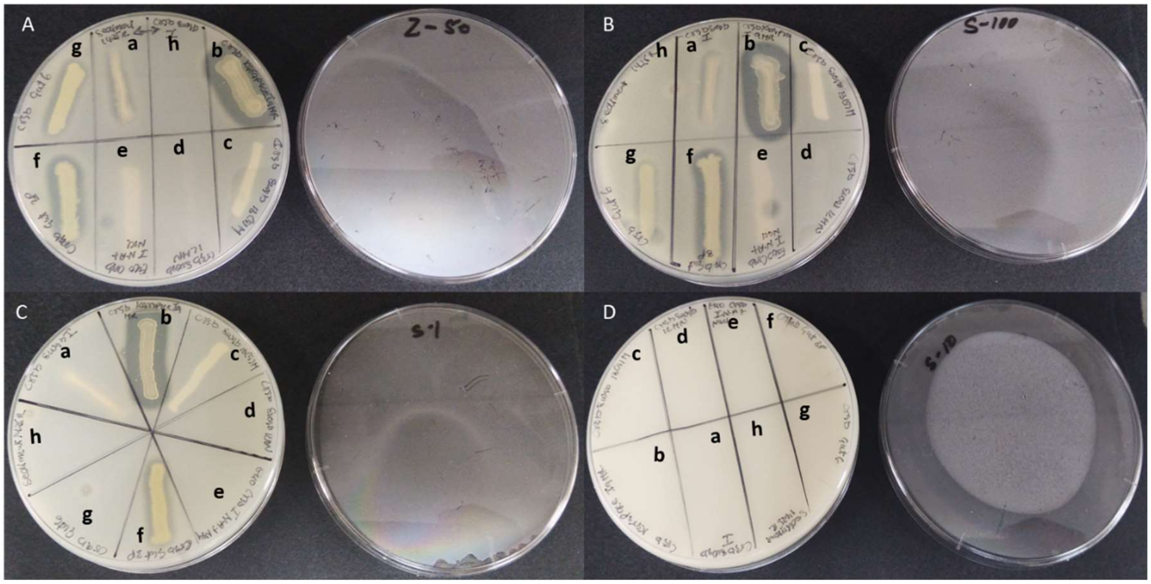

Figure S1. Protease assay in the presence of 50 µM $ZnSO_4$. B. Protease assay with 100 µM $ZnSO_4$. C. Protease assay with 1 mM $ZnSO_4$. D. Protease assay with 10 mM $ZnSO_4$ (*a. Bacillus licheniformis,* b. *Bacillus amyloliquefaciens, c. Staphylococcus haemolyticus, d. Bacillus jeotgali, e. Bacillus firmus, f. Bacillus aquimaris, g. Micrococcus luteus, h. Bacillus subterraneus.*
